# Transient Mild Hyperthermia Induces E-selectin Mediated Localization of Mesoporous Silicon Vectors in Solid Tumors

**DOI:** 10.1371/journal.pone.0086489

**Published:** 2014-02-18

**Authors:** Dickson K. Kirui, Juahua Mai, Anna-Lisa Palange, Guoting Qin, Anne L. van de Ven, Xuewu Liu, Haifa Shen, Mauro Ferrari

**Affiliations:** 1 Department of NanoMedicine, Houston Methodist Research Institute, Houston, Texas, United States of America; 2 Department of Cell and Development Biology, Weill Cornell Medical College, New York, New York, United States of America; 3 Department of Internal Medicine, Weill Cornell Medical College, New York, New York, United States of America; Argonne National Laboratory, United States of America

## Abstract

**Background:**

Hyperthermia treatment has been explored as a strategy to overcome biological barriers that hinder effective drug delivery in solid tumors. Most studies have used mild hyperthermia treatment (MHT) to target the delivery of thermo-sensitive liposomes carriers. Others have studied its application to permeabilize tumor vessels and improve tumor interstitial transport. However, the role of MHT in altering tumor vessel interfacial and adhesion properties and its relationship to improved delivery has not been established. In the present study, we evaluated effects of MHT treatment on tumor vessel flow dynamics and expression of adhesion molecules and assessed enhancement in particle localization using mesoporous silicon vectors (MSVs). We also determined the optimal time window at which maximal accumulation occur.

**Results:**

In this study, using intravital microscopy analyses, we showed that temporal mild hyperthermia (∼1 W/cm^2^) amplified delivery and accumulation of MSVs in orthotopic breast cancer tumors. The number of discoidal MSVs (1000×400 nm) adhering to tumor vasculature increased 6-fold for SUM159 tumors and 3-fold for MCF-7 breast cancer tumors. By flow chamber experiments and Western blotting, we established that a temporal increase in E-selectin expression correlated with enhanced particle accumulation. Furthermore, MHT treatment was shown to increase tumor perfusion in a time-dependent fashion.

**Conclusions:**

Our findings reveal that well-timed mild hyperthermia treatment can transiently elevate tumor transport and alter vascular adhesion properties and thereby provides a means to enhance tumor localization of non-thermally sensitive particles such as MSVs. Such enhancement in accumulation could be leveraged to increase therapeutic efficacy and reduce drug dosing in cancer therapy.

## Introduction

Inadequate delivery of therapeutic agents into solid tumors remains a challenge partly due to biological barriers that hamper effective transport [1]. Efforts directed at overcoming these barriers have included the administration of enzymes to ablate tumor stromal components [2], inhibitors to normalize vessels [1], and application of hyperthermia [3] to reduce tumor density by cell killing. Hyperthermia has widely been used to synergize cancer treatment [4] in experimental animals [5] and in patients [6] where it enhances therapy by direct cell killing, sensitizing cells to chemotherapy [7], radiotherapy [8], and by promoting tumor re-oxygenation [9]. Most of these studies have used high photon flux (5–48 W/cm^2^) to generate thermally ablative temperatures (46–50°C) [7] that kill cancer cells by DNA denaturation [10]. While ablative hyperthermia has proven effective in improving therapy [11], the fact that it causes irreversible tissue damage (including vascular occlusion) represents a major clinical drawback [12]. Patients are often given chemotherapy infusions in multiple cycles for which intact vasculature is necessary.

In the present study, we evaluated the use of low photon laser flux (∼1 W/cm^2^) to generate mild hyperthermia (MHT) to transiently alter vascular adhesion and perfusion properties and thereby amplify particle localization while avoiding vessel occlusive damage. We found a transient increase in vascular-associated adhesion molecules after a temporary mild hyperthermia. MHT (41–42°C) has mostly been used to achieve site-specific delivery of thermally sensitive liposomes [13], [14] and to permeabilize tumor vessels and allow increased therapeutic penetration [15], [16]. Heat stress has also been shown to increase protein expression such as adipose differentiation-related protein (*adfp*) associated with lipid droplet formation in mouse testes [17]. Therefore, we reasoned that heat treatment might also affect a host of other proteins that are vital for particle localization.

Localized MHT treatment have previously been generated using water-bath heating [18], a process that is very slow [19]. Here, we used near infrared (NIR) irradiation combined with tumor-localized gold nanorods (GNRs) to rapidly generate sustained MHT. GNRs were chosen due to their high conversion efficiencies [3] which means that a small amount of particles and low laser power is required to achieve rapid selective heating. After accumulation of stealth GNRs by Enhanced Permeation and Retention (EPR effect) [20], we generated MHT (42°C for 20 min) and evaluated effect of treatment on particle flow and accumulation in two breast cancer tumor models using intravital microscopy (IVM). We chose to create MHT conditions at 42°C for 20 min so as to stay below threshold settings reported to cause permanent vessel occlusive damage known to occur once temperature reaches 43°C and maintained for 240 min [21]. Sustained treatment was given to overcome thermo-tolerance known to occur in cancer cells [22]. Time-lapse tumoritropic particle accumulation was then assessed and correlated to alterations in tumor perfusion, induction of vascular-associated adhesion proteins, and their respective cell-to-particle interactions after MHT treatment.

Effect of mild hyperthermia treatment on accumulation was assessed using multistage mesoporous silicon vectors (MSVs) which are discoidal-shaped particles (1000×400 nm) engineered to adhere onto tumor vessels and form drug depots for sustainable release [23], [24]. Recent efforts have loaded these particles a multitude of chemotherapeutic agents including paclitaxel [25], docetaxel [26], and siRNA-containing liposomes [27]. Preferential localization of MSVs has relied on modulating geometrical properties, with much as 5–8% of injected dose reaching the tumor when discoidal-shaped particles (1000×400 nm) are used [28]. This shape configuration has been determined to favor vascular adhesion and preferentially accumulate in tumor based on in silico mathematical modeling [29] and confirmed *in vivo* [28]. Other strategies employed to increase tumor accumulation have included the use of targeting moieties such as RGD peptide to preferentially target tumor endothelium [28] [30] and E-selectin aptamers to target MSV delivery to bone [31]. MSVs have also been coated with biomimetic cell membranes to produce leuko-like particles with demonstrated improvements in immuno-capture evasion, increased circulation, and tumor accumulation [32].

We studied the effect of MHT treatment on tumor adhesion properties as a strategy to amplify MSV particle localization beyond and above enhancements achieved by geometrical modulation alone. Our study demonstrated that well-timed transient MHT treatment effectively primed the tumor microenvironment, elevated E-selection adhesion molecules which aided amplification of MSV accumulation in tumor. This strategy should open further avenues towards using mild hyperthermia to increase vascular localization of drugs, reduce dosing, and improve therapeutic efficacy.

## Materials and Methods

### Ethical statement

All animal experiments were approved by The Houston Methodist Institutional Animal Care and Use Committee guidelines (Houston, TX) and were performed in accordance to IACUC-approved protocols AUPs 1010-0029 & 1210-0043 and IVM imaging performed under protocol AUP 0611-0032.

### Materials

Fluorescein isothiocyanate (FITC-) and tetramethyl rhodamine isothiocyanate (TRITC-) labeled dextran dyes (70 kDa FITC-dextran), Alexa Fluor 555, and carbocyanine DiD were purchased from Invitrogen (Carlsbad, CA); TNF-α was purchased from Sigma (St. Louis, MO); anti- HSP20, HSP70, vWF, ICAM-1, and E-selectin (CD62E) were purchased from Abcam (Cambridge, MA). All organic solvents used were of analytical grade and used as received. Human cancer cell lines, SUM159 and MCF-7, were obtained from American Tissue Culture Collection (Manassas, VA) and HUVEC cells were obtained from PromoCell (La Jolla, CA). Cell lines were cultured in Dulbecco's modified Eagle's medium (DMEM) (Mediatech, Inc., Manassas, VA) supplemented with 10% (v/v) FBS (Sigma Aldrich, St. Louis, MO) while HUVECs were cultured in Endothelial cell culture medium (PromoCell, La Jolla, CA). All cell lines were grown at 37°C in a humidified incubator containing 5% CO_2_.

### Preparation of fluorescent mesoporous silicon vectors

Discoidal MSV particles (1000×400 nm) were fabricated in the Microelectronics Research Center of The University of Texas at Austin and then fluorescently labeled by reacting them with Alexa Fluor 555 ™ succinimidyl ester as described previously [28]. In brief, 67 µL of Alexa Fluor solution (1 mg/mL, DMSO) was added to a 40 µL suspension of APTES-modified MSVs in 100 mM of triethanolamine (10^9^ particles, DMSO). The mixture was briefly sonicated and gently mixed for 2 h at room temperature under light protection before excess dye was washed out by repeated centrifugation (8,000 rpm, 10 min), vacuum-dried, and then re-suspended in saline buffer (100 µL). These particles were used for IVM and flow chamber studies.

### Generation of breast tumor models

Human-derived breast cancer cell lines were selected to study the effect of MHT treatment. Orthotopic models from SUM159 and MCF7 cell lines were established for these studies. SUM159 breast cancer line is derived from a primary tumor that is highly vascularized while MCF7 exhibits features of differentiated mammary epithelium and is significantly less vascularized than the SUM159. Tumor models were established in Athymic nude mice (Charles River Laboratories, Wilmington, MA) by a one-time injection of tumor cells/Matrigel 1∶1 mixture into the mammary fat pad region. For MCF-7, 17β-estrodiol 60-day release pellet (Innovative Research of America, Sarasota, FL) was subcutaneously implanted 7 days prior to mammary fat pad injection of 5×10^6^ cells.

### Generation of mild hyperthermia

Once tumors reached 5–7 mm in diameter (290–350 mm^3^), animals were injected with 10 mg/kg GNRs and allowed to accumulate in tumor by EPR effect prior to laser irradiation. The concentration of administered GNRs was previously determined to generate desired temperature elevation under NIR irradiation [15]. Initially, GNRs with 810-nm optical absorbance were synthesized and modified with polyethylene glycol (PEG) where PEGylation was used to produce stealthy particles that can passively accumulate in tumor via EPR effect [33]. A detailed protocol used to synthesize and characterize *in vivo* bio-distribution of GNRs is shown in the Supplementary information (Method S1). Tumor-bearing mice was injected with PEGylated GNRs (10 mg GNRs/kg body weight), after 72 h of circulation and tumor passive localization, tumors were focally irradiated with NIR laser to attain 42°C and sustained for 20 min. NIR laser focal heating was chosen due to its deep tissue penetration capabilities (up to 1 cm), rapid heating, and low tissue attenuation [34]. The NIR heating approach is described elsewhere [15] and involved sweeping a 4-mm spot size laser hand-piece across entire tumor surface (∼1 W/cm^2^, Delta 30, Angio-dynamics, UK) for the duration of treatment. Temperature microprobes (Oxford Optronics, Oxford, UK) were used to monitor temperature changes during treatment at the tumor bed as shown in Supplementary information (Figure S1).

### Evaluation of tumor flow dynamics by intravital microscopy

Transient effects of MHT treatment was assessed by evaluating the accumulation of rhodamine-labeled MSVs in two breast cancer tumor lines using intravital microscopy. We also visualized and evaluated changes in blood flow dynamics and established relationships with MHT treatment [28], [35]. For IVM imaging, tumors were exposed using a skin- flap procedure as previously described [28]. Briefly, a midline abdominal incision was performed and a wetted cotton applicator was used to peel away skin and expose the tumor while avoiding severage of tumor vessels. To assist monitor tumor flow dynamics, a one-time injection of fluorescently labeled autologous red blood cells (RBCs) was given 1–2 days before imaging. RBCs were collected by retro orbital bleeding, stained with DiD dye at 37°C using the manufacturer's recommended protocol, and immediately re-injected behind the contralateral eye. Approximately 3–5% of the total RBCs were labeled per mouse [36]. Anesthetized animals were placed and imaged on an upright Nikon A1R MP-ready laser scanning confocal microscope platform equipped with a resonance scanner, isoflurane anesthesia system, heated stage, and custom coverslip mounts [36]. Before imaging, a bolus injection of 70 kDa FITC-dextran (50 µL in PBS) was used to delineate the vasculature. Images were obtained with a three-channel setup in which fluorescence was collected at 488/525 nm for FITC-dextran, at 561/579 nm for rhodamine-labeled MSVs, and at 624/665 nm excitation/emission filters for DiD-labeled RBCs.

Image acquisition was performed over selected field of views (FOVs) with resolution of 512×256 pixels with an optical slice thickness of 7.1 µm. At different time-points after MHT treatment, animals were injected with 5×10^8^ particles (1000×400 nm, 50 µL PBS) and monitored over 60 min after injection. Camera and acquisition settings were kept constant across animals and treatment groups.

### Assessment of vascular perfusion after MHT treatment

The effect of MHT treatment on tumor perfusion was evaluated based on changes in arteriovenous transit time (AVTT) of first-pass flow of dextran tracer. Real-time videos was acquired at 30 frame per second (fps) using a 4x magnification objective lens (3.2×3.2 mm) on IVM. The videos were analyzed as follows: stills were extracted at ten-frame intervals and aligned relative to each other, yielding motion stabilized videos [37]. Regions-of-interest (ROIs) were randomly placed inside vessel segments between branching points, ensuring that there was at least 1 pixel of space between ROIs and vessel margins. Multiple arterioles, venules, and capillaries (10–200 µm diameter) representing the full spectrum of flow dynamics were selected for analysis with ∼30 ROIs per video. The mean fluorescence intensity of each ROI was measured and plotted as a function of time and used to calculate AVTT which is defined as the time difference (delay in perfusion) between venous and arterial flow. We calculated these values using the time it takes to reach ½ maximum fluorescence intensity (half-life) [38]. This method has recently been used to study and describe transport (perfusion) in xenografted human breast cancer models [37]. We expected to obtain different AVTT values if MHT treatment altered tumor perfusion. All IVM quantifications were performed using Nikon NIS Elements v4.0 software.

Changes in tumor flow dynamics resulting from mild hyperthermia was further evaluated by measuring blood velocity based DiD-labeled red blood cell (RBCs). RBC trajectory and velocity through vessels of interest was measured on a frame-by-frame basis to determine the average speed of RBC passage along a 200-µm segment of vessel centered on each adherent particle. RBCs velocity was evaluated on selected FOVs on videos acquired at 30 fps using 4x objective lens with 3x digital zoom [35]. Blood velocity, vessel diameter, and wall shear rate measurements across vessels size (10–200 µm) were obtained using a minimum of 100 RBCs per treatment group and plotted as a distribution by treatment groups. Wall shear rate γ was calculated by assuming laminar flow of Newtonian fluid through a tube: γ = 8ν/d, where ν is the blood flow velocity and d is the vessel diameter. The inner vessel diameter determined from images of intravascular FITC-dextran.

### Assessment of particle-endothelial cell interactions

We assessed potential effect of MHT treatment on vascular endothelial lining and interfacial particle interactions by performing flow chamber experiments, as described previously, using human umbilical endothelial cells (HUVECs) pretreated with MHT [29]. In brief, HUVEC cells were grown to confluence cells in fibronectin-coated cover slip, heated to 42°C for 20 min in a humidified incubator, treated with TNF-α for 6 h (25 ng/mL), and rinsed in PBS. At different time-points after treatment, endothelial monolayer was assembled with a parallel plate flow chamber (GlycoTech Co, Leland, NC), comprised of a PMM flow deck and a silicon rubber gasket. The two components were held together by pulling a vacuum suction using a 3-mL syringe. The inlet and outlet bores on the flow deck were connected using sylastic tubing to allow flow of injected solution in and out of the flow chamber. The flow chamber was mounted on a microscope stage, and an inlet bore attached to 2 mL of MSV particles (10^7^/mL in PBS), and placed on a syringe pump (Harvard Apparatus, MA). Particle flow and interaction with HUVEC cells were monitored on a Nikon Ti- Eclipse inverted microscope (x20 objective) on a bright field channel and TRITC channel used to visualize cells and rhodamine-labeled MSVs, respectively. Sonicated MSVs were injected in a single-pass at a flow rate of 64.516 mL/min, equivalent to 10 s^−1^ which is a relevant shear rate for tumor microcirculation (<100 s^−1^) [29]. Once MSVs injection was completed, unattached particles were washed with injection of 1 mL PBS. Acquired videos were analyzed by enumerating the number of adherent MSVs which was normalized to the injected dose and imaged area on flow chamber. As control experiments, HUVEC cells treated with MHT treatment and incubated anti-E-selectin or -ICAM-1 antibody (2 µg/mL) was used to assess the effect of each adhesion molecule on particle adhesion to endothelial cells.

### Western blotting analysis

The differential expression of vascular-associated adhesion molecules, specifically E-selectin and ICAM-1 that are highly expressed in inflamed tissue we evaluated after time-points after MHT treatment [39]. We also stained for von Willebrand factor (vWF), an adhesive glycoprotein that plays a role in hemostasis and heat shock proteins (HSP) 20 and 70 [40]. In brief, HUVEC cells were grown to confluence and treated with MHT in a humidified incubator and incubated up to 24 h post-treatment. Non-adherent HUVEC cells were washed away while adherent cells were incubated with lysis buffer for 20 min and prepared for Western blotting. Samples contained 30 µg of total protein were electrophoresed and transferred to a nitrocellulose membrane. The membranes were blocked (5% milk) and incubated with primary antibodies against ICAM-1, E-selectin, vWF, HSP20 & HSP70. Membranes were then washed and incubated for 1 h with a horseradish perodixase conjugated anti-rabbit IgG secondary antibody. Proteins were detected using ECL kit.

### Image analyses and particle quantification

The average number of MSVs adherent to tumor vasculature was enumerated in video stills using Nikon NIS element AR software (Nikon, Mellville, NY). Select FOVs were chosen from time-lapse videos and automated object measurement feature was used to count the number of fluorescent particles in each frame where a particle was defined by setting low and high pixel thresholds to include only visible red fluorescent particles and to exclude single noise pixels. The settings were applied to all frames and automated counting function used to generate total particle count for each time point up to 60 min. We manually counted particles to confirm threshold settings used for automated particle enumeration. The average number of particles was normalized to the imaging volume and then plotted as a function of time. Representative video from which acquisition was performed is provided in the Supplementary section.

Tumor perfusion was characterized using first-pass video stills of a bolus 70-kDa dextran dye injection from which time-lapse fluorescent intensities were extracted, plotted as a function of time, and then used to calculate AVTT across the various treatment groups. Further perfusion analyses using RBCs were performed in which respective flow velocities through vessels were measured using automated tracking options on Nikon NIS element AR software. Threshold pixels were correctly adjusted to identify and track movements of single RBC across 3–5 continue frames. The average velocity in each vessel and size were measured and used to calculate the average shear rate. The effect of MHT treatment on tumor flow and perfusion was evaluated by comparing changes in RBC velocities and shear rates.

### Statistical analysis

All data are presented as means ± standard error of the mean (SEM). GraphPad statistical software (La Jolla, CA) was used to determine statistical significance between treatment and control groups using student's test and one-way ANOVA test was used to compare difference between groups. A value of p <0.05 was considered statistically significant.

## Results

### Near infrared irradiation creates sustained mild hyperthermia

Sustained localized hyperthermia treatment can be achieved using several technologies including high focused ultrasound [41] and near infrared irradiation [6], [42]. We chose to use NIR irradiation because it can penetrate deeply into tissue (up to 1 cm) [34] and produces selective heating when combined with localized GNRs that possess high-energy conversion efficiencies (>10^4^ higher than common fluorochromes) [3]. We have previously reported the synthesis and modification of these PEG-coated GNRs that make stealth particles amenable to passive EPR-mediated tumor accumulation [15]. *In vivo* bio-distribution of PEG-coated GNRs analyses showed that 150 µg GNRs/g of tumor (∼13% of injected dose) accumulated in SUM159 breast tumor and 103 µg GNRs/g of tumor (9%) for MCF-7 tumor. Majority of these GNRs were eliminated from circulation by splenic clearance after 72 h (Figure S1).

When orthotopic tumors were irradiated with pulsed NIR laser (∼1 W/cm^2^), MHT treatment was rapidly attained with tumor temperature reaching ∼42°C at the base and sustained for 20 min (Figure S2). This approach achieved desired temperature (42°C) within 90 min reach 42°C [19]. While previous studies have demonstrated the use of hyperthermia to accumulate particles by induction innate inflammation and coagulation used to drive particle accumulation [43], these strategies, however, require the use of ablative, hyperthermia which are only suitable for one-time enhanced delivery. Ablative hyperthermia cause vascular occlusion and would be un-realistic approach in clinical settings given that treatments are typically administered through multiple rounds of drug infusion. We generated MHT that was steadily sustained for a prolonged period without significant increase in cell apoptosis (TUNEL assay). In contrast, ablative hyperthermia increased the proportion of apoptotic and necrotic positive cell population (Figure S2).

### IVM imaging allows dynamic monitoring of particle flow in tumor

**Figure 1:**
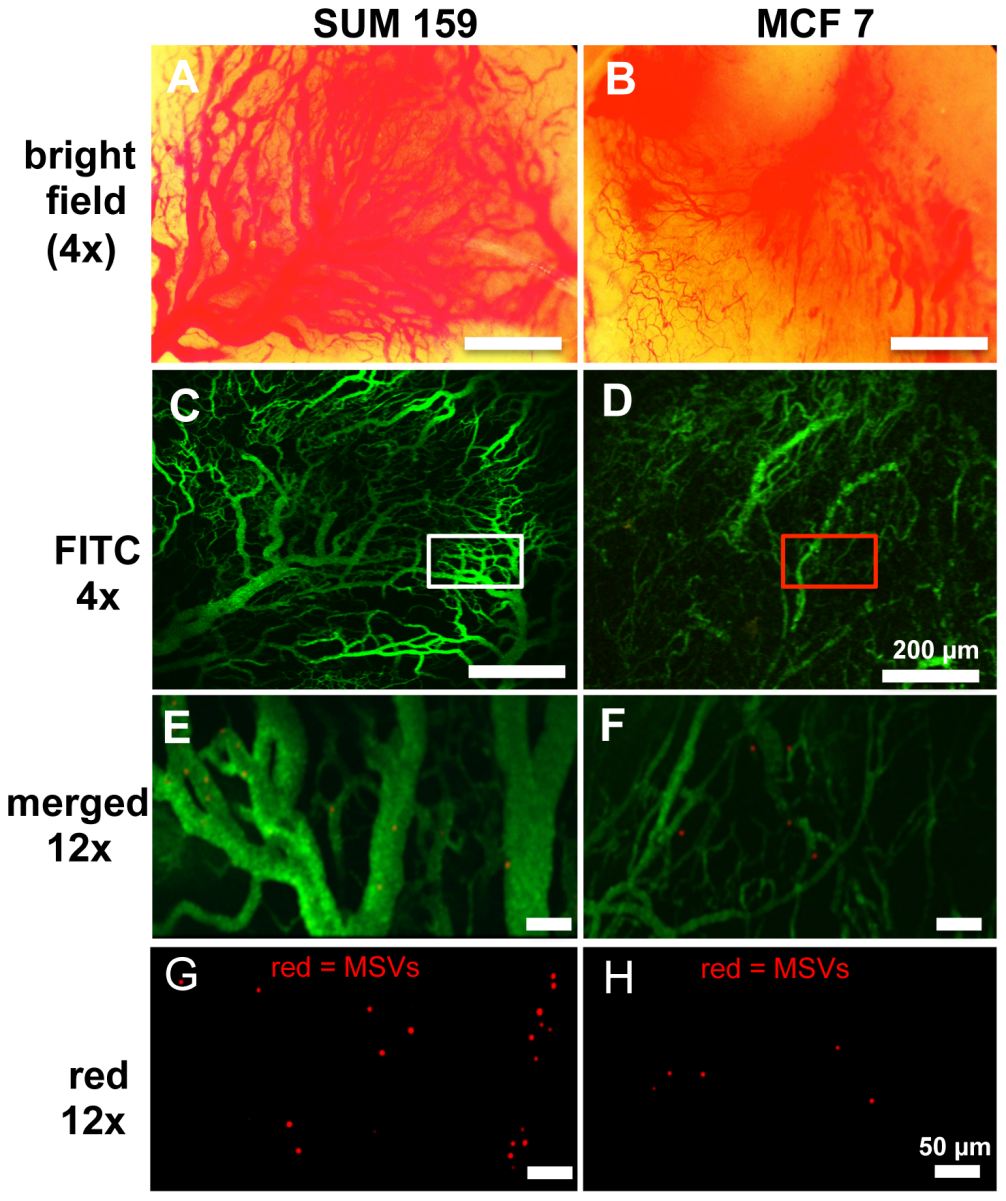
Visualization of tumor particle dynamic flow by intravital microscopy. A, B) Wide-field images of SUM159 and MCF7 tumors exposed via skin-flap. SUM159 tumors are characterized by highly dense network of dilated vessels whereas MCF7 tumors are mostly comprised of smaller, more widely spaced vessels; C, D) Tumor vasculature was delineated following bolus *i.v.* injection of 70 kDa FITC-dextran; E-H) At higher magnification, individual rhodamine-labeled MSVs were readily identified. Merged images (E, F) show particle localization relative to the tumor vessels 60 sec after particle injection.

The effects of MHT treatment on tumoritropic particle flow and accumulation were visualized and evaluated by IVM. Figure 1 shows characterization of two breast cancer tumor lines selected due to their distinctly different vascularization (Figure 1 A–D). We chose to study the effects of MHT treatment in these two breast tumor models in order to establish differences in particle accumulation in tumors of varying vascular density. A bolus injection of FITC-dextran tracer revealed well-formed, dense vasculature in SUM159 tumor and lesser dense network in MCF-7 (Figure 1C-F). IVM visualization allowed exquisite delineation of vasculature region with fluorescent tracer and on other channels, monitoring flow and accumulation of rhodamine-labeled MSVs in vessels of varying sizes and density (Figure 1G, H). The use of IVM in this study enabled us to also resolve flow of individual particles through vessels (10–200 µm) and to monitor accumulation at various time-points after MHT treatment (representative video is available in Video S1).

### MHT increases tumoritropic particle flow and accumulation

**Figure 2:**
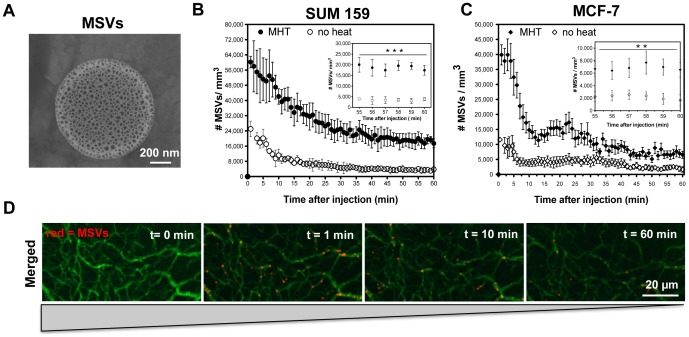
Mild hyperthermia treatment enhances particle inflow and localization in breast tumor models. A) SEM micrograph of porous MSVs (1000×400 nm) that allow therapeutic loading; B, C) Time-lapse quantification analyses of fluorescently-labeled MSVs in tumor vasculature showing increased initial and total number MSVs in tumors receiving MHT treatment versus control groups. 5-fold enhancement was observed in SUM159 and 3-fold for MCF7; D) Representative time-lapse images from videos acquired over 60 min, showed decreased number of MSVs with time and adherent particles shown after 60 min. Curves represent number of particles monitored in animals (n = 6) injected with 5×10^8^ particles (1000×400 nm, 50 µL PBS) and monitored over 60 min. Error bars represent standard deviation from (n = 6) collected over 8 FOVs per animal. Statistical significance between treatment and control group was determined based 55–60 min time-points after injection where *** show p<0.006 and ** show p<0.02.

Transient effects of mild hyperthermia and its potential uses to augment particle accumulation were evaluated by measuring the accumulation of MSVs. These particles, shown in Figure 2 A, are biodegradable drug carriers that have been packaged with a multitude of therapeutic agents [44] and are envisioned as drug depots that lodge onto tumor vasculature [45]. IVM was used to assess the effect of MHT treatment on MSVs accumulation in tumor microenvironment. Initially, we evaluated the effect at a single time-point. MSV accumulation was monitored in real-time for 60 min after injection where tumor-receiving MHT treatment showed more than 2-fold increase in the initial number of particle flowing into tumor compared to SUM159 control group (Figure 2 B). MSV clearance from bloodstream is rapid [28], [36], resulting in time-dependent decrease in the number of circulating particles. Thus, increased particle flow suggested that MHT treatment altered tumor flow dynamics. The total number of particles remaining in tumor microenvironment at 60 min post-injection remained unchanged for both treated and control groups, and thus elected this time-point to evaluate the proportion of MSVs adherent to tumor vasculature. At this point, most of the circulating MSVs had been cleared (Video S2).

Based on these analyses, MHT treatment led to ∼6-fold enhancement in the cumulative number of adherent MSVs compared to untreated group (Figure 2 B). Similar enhancement in number of particles flowing into tumor (∼3.5-fold) and cumulative adherent MSVs (3-fold) was observed for MCF-7 tumor model (Figure 2 C). Representative IVM images of MSVs (red) flow and adherent MSV onto vasculature at 60 min are shown in Figure 2 D while video illustrating particle flow and accumulation in SUM159 tumor is shown in (Video S2). This finding suggested that MHT treatment augments accumulation particles (MSVs), independent of tumor model and tumor vascularity. SUM159 is a highly vascularized tumor model (45% of total area) than MCF-7 (12%) as was quantified by ImageJ® software, see the Supplementary information (Method S2) and (Figure S3).

### MHT treatment induces a time-dependent augmentation in MSVs accumulation

**Figure 3:**
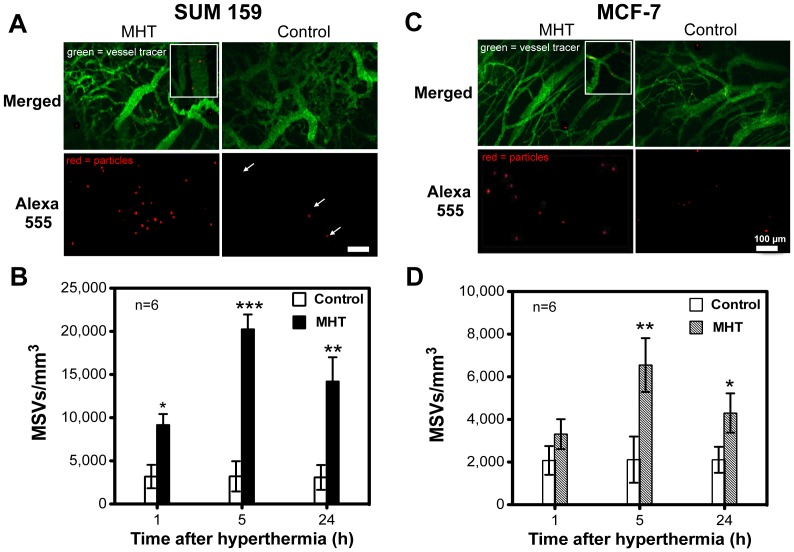
Mild hyperthermia treatment enhances MSV accumulation in a time-dependent manner. A) Representative IVM tissue images of SUM159 tumor show higher accumulation in tumors receiving MHT when analyzed 5 h after treatment; B) quantitative analyses time-dependent increase in accumulation with ∼6-fold increase at 5 h and a reduction in enhancement at 24 h post-treatment (3-fold); C) Histological IVM image illustrating increased MSVs in MCF-7 (less vascularized cell line); D) Quantitative analyses showed similar time-dependent increases in MCF-7 which abated at 24 h. At each time-point, MSVs were injected and allowed 1 h to circulate before analyses. Error bar represents replicates of n = 6, with statistical significance denoted by *** p<0.0001; ** p<0.005; ** p<0.01 for SUM159 and ** p<0.0045 and * p<0.02 for MCF-7 line.

We next evaluated time-dependent MHT effect and MSV accumulation to obtain the optimal time of opportunity at which MHT treatment can effectively be used to enhance tumoritropic localization. For this study, 60-min time-point was chosen to assess the proportion of adherent particles based on previous finding. MSVs accumulation was evaluated after treatment (up to 24 h) versus controls and also compared to findings obtained from dynamic flow (Figure 2). MSV particles were injected at multiple time-points after MHT treatment using separate animals to analyze accumulation at each time-point where mouse-to-mouse variation were minimized by keeping acquisition parameters same. Histological images illustrating increased accumulation of MSVs across two tumor models shown in Figure 3A, C. By visual inspection, majority of MSVs in both models appeared adhered to vascular walls and at a higher rate in tumors receiving MHT treatment. This suggested that MHT treatment affected vascular endothelium binding properties and interactions with MSV particles (Figure 3 A, C). Quantitative analyses showed a slight increase in MSV accumulation at 1 h and maximal particle augmentation (6-fold increase) at 5 h time-point while the MHT effect appeared to diminish at 24 h after treatment. Similar enhancements in particle accumulation were observed for MCF-7 receiving MHT treatment with ∼4-fold increase at the 5 h time-point compared to control group (Figure 3 C, D). At 24 h, the effect was still statistically significant compared to untreated control but abated relative to the effect at 5 h after treatment. While fewer particles flowing into tumor was observed for MCF-7, enhancement of MSV accumulation was still evident at various time-points. At 1 h post-treatment, there was a slight increase in enhancement, maximal augmentation at 5 h time-point, and a reduction in enhancement at 24 h after treatment. The observation of time-dependent particle accumulation, i.e. maximal MSV accumulation at 5 h and abatement at later time-point, illustrated that this strategy creates a transient a window-of-opportunity with which to increase particle delivery. This suggested that a combination of MHT tumor priming could be optimally used to synergize the delivery of chemotherapeutic-loaded particles such as MSVs.

MHT treatment alters tumor perfusion with proportional increase in MSV localization

**Figure 4:**
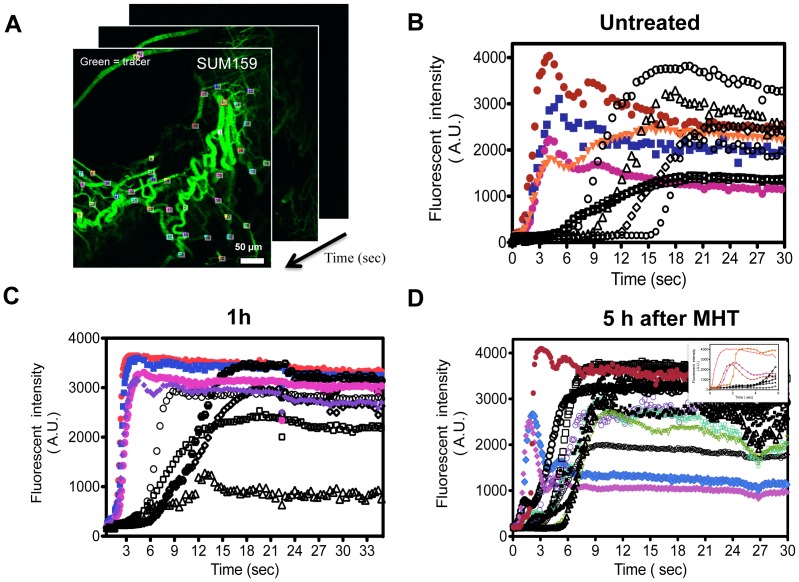
MHT treatment increases tumor dynamic flow as characterized by dye perfusion. A) Representative ROIs selected for time-lapse fluorescence analyses in SUM159 tumor. ROIs were randomly defined inside arterioles, venules and capillaries, between branching points, yielding approximately ∼40 ROIs per video; B) Perfusion curves of untreated SUM159 tumor with arterial (colored) and venous perfusion (black) characterized by rapid arteriole flow and delayed venous flow; C) Altered perfusion plots of MHT treatment group characterized by steeper and more rapid arteriole flow and shortened venous flow; D) tabulated summary shows by shortened dextran half-life and AVTT where ± represent standard deviations in ∼120 ROIs from n = 6 with statistical significance denoted by * p<0.033 at 5 h after treatment relative to untreated group. The videos from which the data have been derived are found in supplementary data.

Initial observation indicated increased particle flow into tumor after MHT treatment, suggesting potential correlation between MHT treatment and tumor perfusion. To confirm this hypothesis, first we evaluated changes in vessel flow after *i.v.* injection of 70 kDa FITC-dextran tracer. The first-pass perfusion was recorded in real-time using IVM at 30 frame per second (fps) and frame-by-frame analysis of tracer intensity was performed on selected ROIs, yielding perfusion time curves. Figure 4 A shows representative time-lapse images acquired immediately after bolus injection of 70 kDa FITC-dextran. Time-lapse dye vessel intensities were used to generate arteriole and venous perfusion plots shown in Figure 4 B, C. The arteriovenous transit time (AVTT), defined as the time difference (delay in perfusion) between venous and arterial flow, was calculated from these plots. Several key features can be observed: For untreated tumor, the arterial curves (colored) are characterized by a rapid increase in fluorescence intensity which plateaus within 15 sec, drops off and levels out. The venous curves (black) show a prolonged and a gradual increase in fluorescence intensity which plateaus within 25 sec and is characterized by delay in perfusion. In normal tissue, this delay is a few seconds and can be a few seconds to a minute for various tumor types [37]. For un-treated SUM159 tumors, the perfusion of arterial system was characterized by rapid rise in fluorescence intensity where it took 5.2±1.4 sec to reach half maximum intensity while venous phase took 15.1±2.6 sec for venous flow and AVTT of ∼10.2±2.7 sec (Figure 4 D). Assessment of perfusion at 1 h post-treatment revealed steeper rate of flow in the arterial system reaching half maximum intensity at 3.4±1.9 sec and a shortened delay in venous flow (9.7±2.4 sec) corresponding to AVTT of ∼6.6±1.3 sec (not shown). Tumor transport phenomena were significantly altered when it was assessed at 5 h after MHT treatment with the arterial perfusion drastically shortened to 2.1±1.3 sec, a venous system of 5.8±2.1 sec, yielding a significant reduction in AVTT (3.4±1.4 sec). At 24 h after treatment, perfusion characteristics appeared to return to basal level with arteriole half-life of 3.4±1.1 sec, venous half-life of 10.2±2.9 sec and AVTT of 6.9±2.2 sec (not shown). A tabulated summary of these results is shown in Table 1 in which there was a statistically significant increase in perfusion at 5 h after MHT treatment. Representative IVM videos of the SUM159 from which these analyses were derived are provided in the supplementary (Video S3).

**Table 1:** Alterations in tumor perfusion after MHT treatment.

Time (h) after MHT				
Parameter	Untreated	1 h	5 h	24 h
**Arterial** (sec)	5.2±1.8	3.4±1.9	2.1±1.0	3.4±1.1
**Venous** (sec)	15.1±2.6	9.7±2.4	5.8±2.1	10.2±2.9
**AVTT** (sec)	10.2±2.7	6.6±2.2	3.4±1.4 *	6.9±2.2
		AVTT = Arteriovenous transit time; n = 5		

Concurrent reduction in arterial and venous flow after with MHT treatment which contributed to decreased half-life AVTT. Tabulated data represents average of ∼30 ROIs per treatment group (n = 5) where * denotes p<0.03.

**Figure 5:**
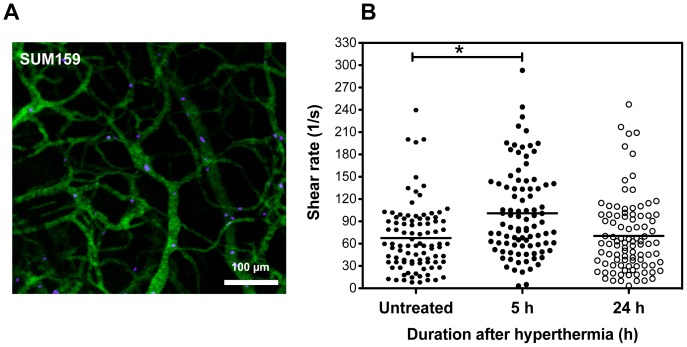
Evaluation of tumor perfusion after MHT treatment in SUM159 tumor using RBCs as surrogate. A) fluorescent dextran labeling of tumor vessels (green) were used to delineate and enable tracking of autologous red blood cells (purple); B) Increased RBCs shear rates with MHT treatment calculated from measured cell velocities and vessel diameter over ∼100 ROIs per treatment group showing increased shear rate at 5 h after MHT treatment. Statically significant increase in shear rate was observed at 5 h after MHT treatment. Each data point represent average velocity of 3 RBCs/per vessel obtained from n = 3 per time-point and * denotes *p*<0.03.

The effect of MHT treatment on tumor perfusion was further corroborated by tracing flow and cell velocity using fluorescently-labeled RBCs as illustrated in Figure 5 A. The average shear rates were calculated, taking into account their respective vessel diameter. Measurements obtained from ∼100 ROIs showed that mean shear rate of RBCs through SUM159 control group was 67.8 s^−1^ which increased to 98.2 s^−1^ at 5 h and trended towards basal level at 24 h time-point (70.1 s^−1^) (Figure 5B). Video demonstrating flow of RBCs (blue) in tumor is shown in the Supplementary (Video S4) while representative movies of individually pseudo-colored RBCs that enabled cell velocity tracking and analyses are also illustrated in the Supplementary (Video S5). These findings showed that maximal perfusion coincided with the highest maximal MSVs accumulation, suggesting a correlation. This pattern of increased perfusion with hyperthermia treatment is consistent with previous findings [37] where a transient increase in perfusion was shown after 12 h after treatment.

### Mild hyperthermia increases MSVs adherence to HUVECs

**Figure 6:**
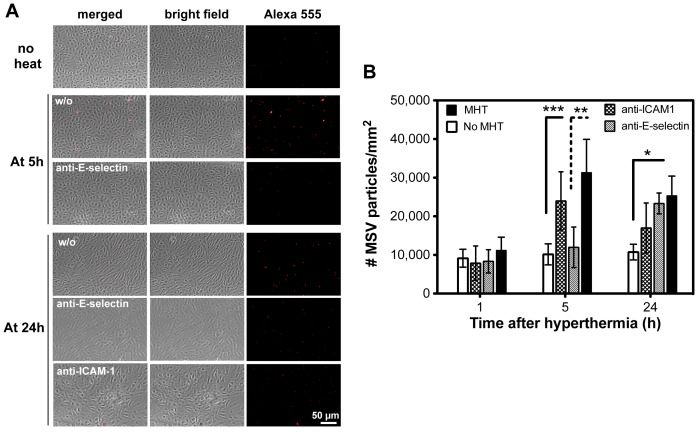
Flow chamber experimentation revealed increased particle-endothelial cell interactions after MHT treatment. A) representative images demonstrate extent of particle-HUVEC cells binding without treatment (top panel); at 5 h, the number of MSVs attached to HUVECs is significantly increased relative to control but the effect was negated upon incubation with anti-E-selectin; at 24 h, MSV binding is significant compared to control and incubation with anti-ICAM-1 slightly reduced binding while anti-E-selectin caused in insignificant reduced; B) Quantitative analyses demonstrating significant increase in particle adhesion to HUVECs cells with highest increase occurring at 5 h. Pre-incubation with anti-E-selectin reduced cell binding to almost basal levels. MSVs are fluorescent labeled (red) with Alexa Fluor 555 in which video and images are acquired with x20 objective lens. Error bars represent s.d. in 30 field of views (3.2×3.2 mm) from n = 3, with statistical significance denoted by * p<0.021, ** p<0.016, and *** p<0.009.

We observed that localized MHT treatment increased MSVs accumulation that were primarily lodged on tumor vasculature in low- and well-vascularized tumors, and therefore hypothesized that hyperthermia increases particle-endothelial cell interfacial interactions. In the next set of experiments, we evaluated the interactions between HUVEC cells and MSVs in flow chamber experiments. Endothelial cells grown on fibronectin-coated cover slips were used to study their interaction with MSVs at different time-points after MHT treatment. Figure 6 A show representative images of MSVs (red) attached to the monolayer of endothelial cells at the end of flow experiments. At 1 h after MHT treatment, HUVEC cell-particle interaction remained relatively unchanged as evidenced by slight and insignificant increase in MSV attachment. However, cell-MSVs interactions were maximal at 5 h after treatment, with the highest number of MSVs attaching to HUVEC monolayer. Upon incubation with anti-E-selectin, this effect was almost completely negated while similar treatment with anti-ICAM-1 had minimal effect on particle binding (Figure 6 A). This result suggested that E-selectin plays a role in anchoring MSV particles onto the endothelial lining during flow. At 24 h after MHT, the number of adherent MSVs were reduced compared to enhancements at 5 h (Figure 6 A, (w/o)). Incubation with anti-E-selectin, conversely, did not alter the number of attached MSVs while incubation with anti-ICAM-1 slightly reduced the extent of MSV attachment (Figure 6 A). Quantitative analyses of adherent MSVs over ∼30 FOVs are shown in Figure 6 B. MHT treatment statistically increased the number of adherent MSVs at 5 h time-point while pre-treatment with anti-E-selectin negated this effect. Enhancement obtained from MHT treatment appeared to abate at 24 h where reduction in the number of enumerated MSVs is notable. The effect of anti-ICAM-1 treatment is more pronounced than effect of anti-E-selectin at the 24 h time-point (Figure 6 B). These experiments correlated increased particle-HUVEC cell association to elevated expression of E-selectin adhesion molecules caused by MHT treatment. It demonstrates that MHT heat stimulation can positively affect particle-cell interaction by induction of vascular-associated adhesion molecules that can mediate particle associations along vessel walls.

### Mild hyperthermia induces expression of vascular-associated adhesion markers

**Figure 7:**
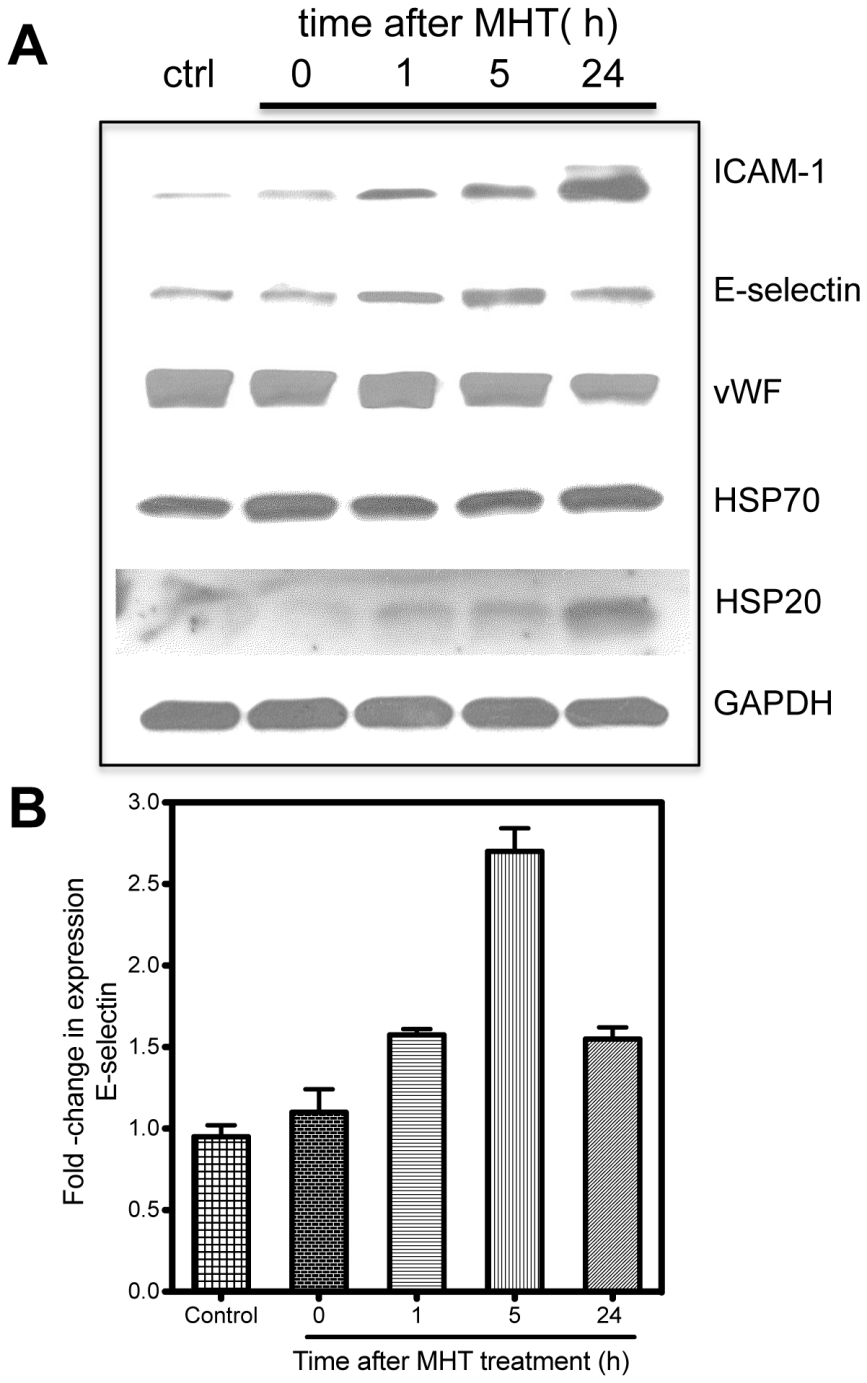
Western blot reveal that MHT treatment induces E-selection and ICAM-1 expression in a time dependence manner. A) Western blotting reveals MHT treatment stimulated E- selection expression which peaked at 5 h and ICAM-1 expression leveling off at 24 h post-treatment while vWF and HSP70 remained un-stimulated by MHT treatment; B) Gel quantification showed increasing expression peaking at 2.5-fold higher expression at 5 h and which abated at 24 h post treatment. Unchanged HSP70 expression and slight increase in HSP20 indicated that MHT treatment caused minimal cell damage. Fold changes in protein expression are normalized to GAPDH levels while error bars represent s.d. from triplicate western blots performed.

Flow chamber experiments suggested a role and differential expression of adhesion molecules that enhanced particle-cell interactions along the tumor vascular walls. Hence, we performed Western blot analyses to evaluate protein expression profiles and establish any correlation with increased binding to HUVEC cells after MHT treatment. E-selectin and ICAM-1 expression were our primary targets since they are highly expressed inflammatory sites and could potentially affect endothelial cell interactions with MSV particles [39]. Western blot analyses showed a linear increase in the expression of E-selectin that reached maximal level at 5 h and decreased at 24 h after MHT treatment (Figure 7 A). On the other hand, ICAM-1 expression linearly increased reaching a maximal expression at 24 h time-point. The expression levels of vWF and HSP70 were un-stimulated, suggesting that MHT treatment caused minimal adverse inflammatory effects (based level on HSP levels). Qualitative blot analyses (ImageJ, NIH) showed that MHT treatment resulted in 2.5-fold increase in E-selectin at 5 h and 1.5-fold at 24 h after treatment (Figure 7 B). Previous work have shown similar expression patterns, with maximal E-selection expression reached at 6–12 h after HUVEC cell activation which trended towards basal levels after 24 h. The expression levels of ICAM-1 upon activation has been shown to be time-dependent, reaching a plateau at 24 h [39].

Together, these findings suggested that E-selectin, whose expression coincided with highest *in vivo* tumoritropic particle accumulation, may play a pivotal role than ICAM-1 in interfacing MSV particles to endothelial cells along the vessel walls. E-selectin expression probably increases the interaction of MSVs to endothelial cells lining the vessel walls and hence enhances the proportion of adherent MSV particles.

## Discussion

Hyperthermia treatment has been shown to improve efficacy of nanomedicine with demonstrated enhancements in accumulation of thermosensitive liposomes [46], [47], tumor targeting [48], and improved tumor penetration [49]. In this study, we evaluated the effect of MHT treatment on tumor vessel dynamics, including perfusion and adhesion properties which, in turn, affected particle localization. NIR irradiation was chosen to generate MHT treatment because it can penetrate deeply into tissue (up to 1 cm) [34] when mediated by GNRs that possess strong optical absorption coefficients [50], [51]. Prior to heating, PEGylated gold GNRs were localized in tumor by EPR effect and used to remotely generate mild tumor heating. This heating strategy achieves the desired temperature faster and deeper in tissue (Figure S2) than convectional water-bath heating, a process that is indiscriminate and slow. MHT treatment was sustained at 42°C so as to avoid occlusive vascular damage which occurs at higher temperature (46–50°C) [52]. While previous studies have demonstrated the use of ablative hyperthermia to accumulate particles by inducing innate inflammation and coagulation used to enhance particle localization [43], these strategies cause vessel damage and would only suitable for one-time enhanced delivery. The approach is un-realistic approach in clinical settings given that treatments are typically administered through multiple rounds of drug infusion. Here, we evaluated the use of MHT treatment to modulate tumor properties and enhance accumulation of MSVs without causing irreversible vascular damage.

With this strategy, we have demonstrated that MHT treatment amplified of particle accumulation in tumor models both with low and high vascularization densities, suggesting effectiveness across a wide spectrum of tumor profiles (Figure 1). Intravital microscopy analyses revealed that hyperthermia alters tumor flow dynamics as evidenced by the drastic increase in particle flow into tumor (Figure 2). MHT treatment also enabled a time-dependent particle accumulation in both tumor models, yielding highest accumulation at 5 h and abating at 24 h after treatment (Figure 3). This suggested that MHT tumor priming is transient and has to be optimally timed in order to synergize the delivery of drug-loaded particles. Enhanced particle accumulation correlated with increased perfusion as evidenced by shortened AVTT values as summarized in Table 1 and increased cell velocities and shear rates (Figure 5). These findings are consistent with previous uses of hyperthermia where increased blood perfusion in normal tissue (twelve-fold) [53] and in breast cancer tumors (two-fold) [12] are reported.

We also demonstrated, using flow chamber experimentation, that MHT treatment increase particle-HUVEC cell interaction in a time-dependent manner, with highest interactions occurring at 5 h and abating 24 h after treatment (Figure 6). To further correlate the particle-HUVEC cell propensity binding to increased induction of adhesion molecule expression driven by MHT, we evaluated the number of particles binding to HUVECs cells after pre-treatment with antibodies against E-selection or ICAM-1. Results demonstrated that the number of adherent MSVs on cells incubated with anti-E-selectin was vastly decreased at 5 h after MHT (Figure 6). Conversely, HUVEC cells treated with anti-ICAM-1 showed significant reduction in MSV attachment at 24 h after treatment (Figure 6). We have demonstrated that MHT treatment can positively affect particle-cell interaction by stimulating the induction of vascular-associated adhesion molecules that mediate associations along vessel walls. Western blot analyses confirmed that MHT treatment stimulated E-selectin expression reaching maximal expression at 5 h and returning to basal levels after 24 h (Figure 7). Meanwhile, ICAM-1 induction increased linearly, reaching its highest expression at 24 h after treatment (Figure 7). Similar patterns of expressions are reported in the literature where maximal E-selection induction was reached between 6-12 h after HUVEC cell activation and trended toward basal levels after 24 h. The expression of ICAM-1 after activation increased has also been shown to increase with time, reaching a plateau at 24 h [39]. Together these findings suggested that E-selectin, whose expression coincided with highest *in vivo* tumoritropic particle accumulation, may play a pivotal role than ICAM-1 in interfacing endothelial cells on the vessel walls with MSV particles. E-selectin expression probably increases the interactions of MSVs to endothelial cells lining the vessel walls and hence enhances the proportion of adherent MSV particles.

## Conclusion

This study demonstrated that well-timed mild hyperthermia treatment can be an effective tumor priming strategy that can transiently elevate tumor transport properties, alter vascular adhesion properties. It provides the rationale towards the deployment MHT treatment as a vital tool with which to synergize delivery and tumor localization of therapeutic-loaded particles. Such efforts would open up more avenues toward MHT-mediated treatment strategies would lead to improved therapeutic efficacy and reduced drug dosing.

## Supporting Information

Figure S1 **Bio-distribution of PEG-coated gold nanorods (GNRs) after intravenous injection and 72 h of circulation in breast cancer tumors.** A) ICP-MS analyses showing tumoritropic reaching ∼13% GNRs of injected dose in SUM159; B) while 9% of injected dose accumulates in MCF-7.
(TIF)

Figure S2 **NIR irradiation generated sustained mild hyperthermia with minimal cellular damage.** A) Laser irradiation generated sustained MHT profile at ∼42°C; B, C) TUNEL assay reveals that MHT treatment caused minimal cell damage in which showed minimal brown apoptotic cell population were observed; D) and compares well to untreated control; E) while ablative hyperthermia resulted in significant cell destruction indicated by significant apoptotic cell population.
(TIF)

Figure S3 **Tumor vascular analyses reveal difference in vascular characteristics in SUM159 and MCF-7 tumor cell lines.** Images acquired by IVM were converted to 8-bits and vascular indices analyzed by ImageJ based on coverage of vascular tracer (70 kDa FITC-dextran).
(TIF)

Method S1 **Synthesis, functionalization, and characterization, and** ***in vivo*** **bio-distribution of gold nanorods used to generate localized MHT treatment.**
(DOCX)

Method S2 **Characterization of tumor cell viability by TUNEL assay and quantification of tumor vascularity with ImageJ®.**
(DOCX)

Video S1 **Representative video showing MSV circulation upon** ***i.v.*** **injection.** Tumor vessels are labeled by bolus injection of 70 kDa dextran (green) which was followed by injection of 5×10^8^ MSVs. Rhodamine-labeled MSVs (red) are seen circulating immediately after injection (less than 10 sec) and monitored for 60 min.
(MP4)

Video S2 **Movie acquired after 60 min of circulation showing adherent MSVs.** Rhodhamine-labeled MSVs (red) are adherent to tumor vessel walls are observed after 60 min. Proportion of adherence increase drastically with MHT treatment.
(MP4)

Video S3 **Video illustration of first-pass tumor perfusion using 70-kDa FITC-dextran.** Changes in tumor perfusion from MHT treatment were quantified by extent of dextran perfusion.
(MOV)

Video S4 **Video shows RBCs circulation in SUM159 tumor microenvironment.** Alterations in tumor perfusion were calculated based in changes in RBC velocities and shear rates through tumor vessels (green). Prior to analyses RBCs were labeled with DiI lipophilic dye resulting in fluorescent cells (blue).
(M4V)

Video S5 **Illustration of RBC flow analyses using NIS elements.** Selected RBCs were pseudo-colored to enable tracking and velocity and shear rate determination.
(M4V)
