# Supplementary figures and images for: Transient Mild Hyperthermia Induces E-selectin Mediated Localization of Mesoporous Silicon Vectors in Solid Tumors
Source: PLoS One. 2014 Feb 18;9(2):e86489. doi: 10.1371/journal.pone.0086489 (PMC3928046; doi:10.1371/journal.pone.0086489)

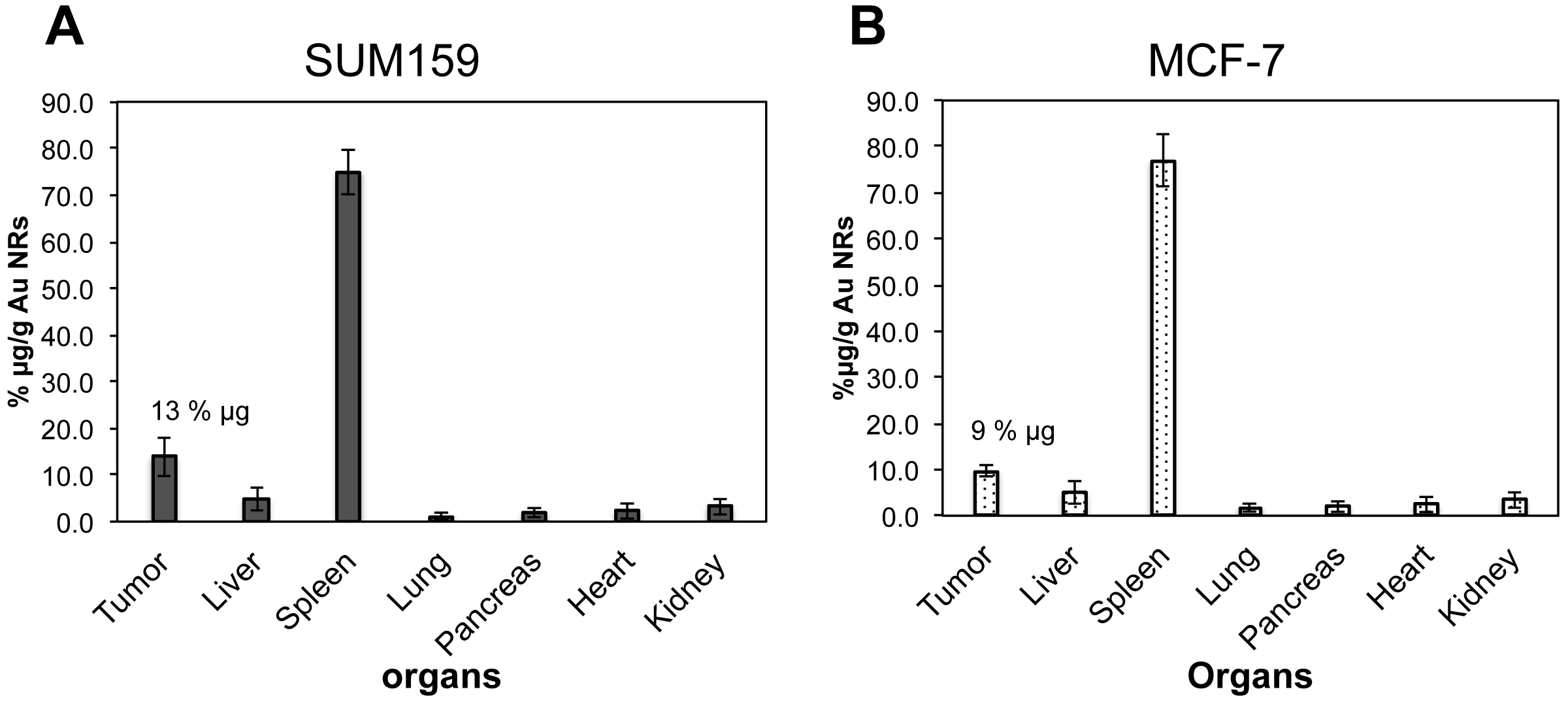

Supplement: Figure S1 — Bio-distribution of PEG-coated gold nanorods (GNRs) after intravenous injection and 72 h of circulation in breast cancer tumors. A) ICP-MS analyses showing tumoritropic reaching ∼13% GNRs of injected dose in SUM159; B) while 9% of injected dose accumulates in MCF-7. (TIF) [file pone.0086489.s001.tif]

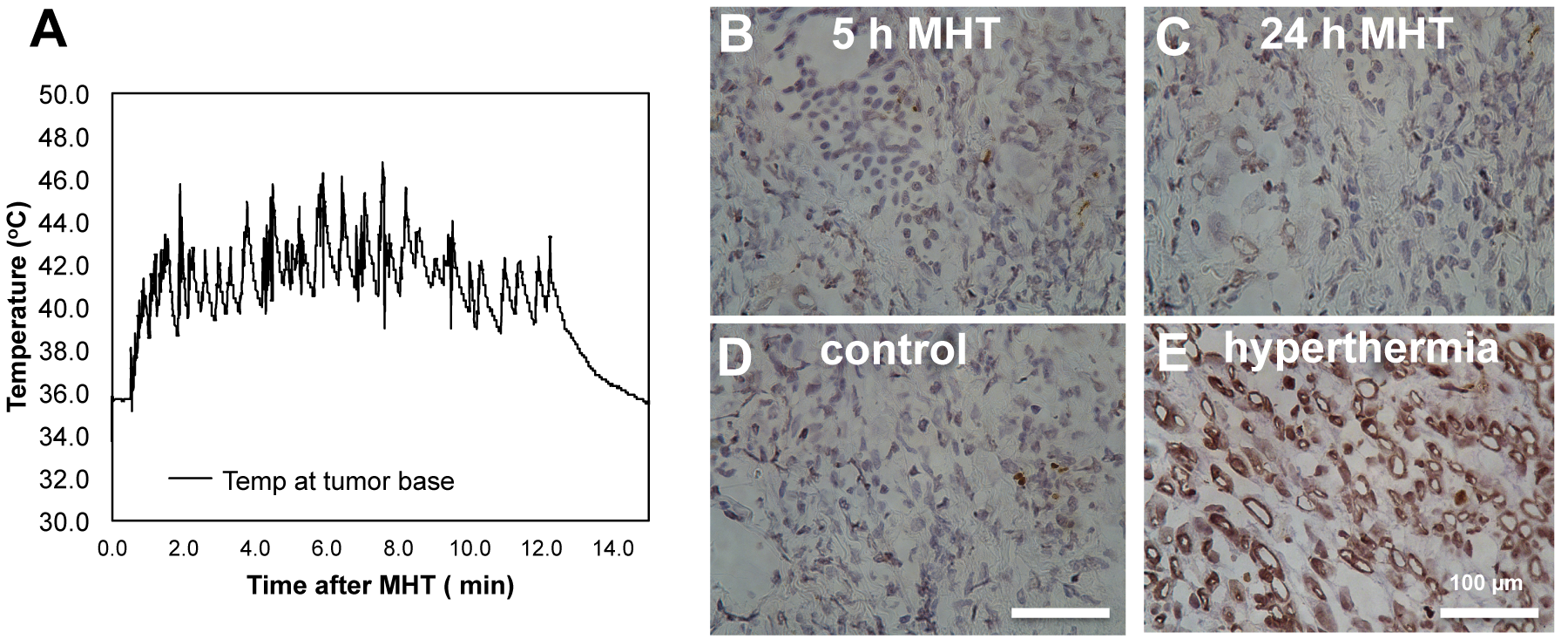

Supplement: Figure S2 — NIR irradiation generated sustained mild hyperthermia with minimal cellular damage. A) Laser irradiation generated sustained MHT profile at ∼42°C; B, C) TUNEL assay reveals that MHT treatment caused minimal cell damage in which showed minimal brown apoptotic cell population were observed; D) and compares well to untreated control; E) while ablative hyperthermia resulted in significant cell destruction indicated by significant apoptotic cell population. (TIF) [file pone.0086489.s002.tif]

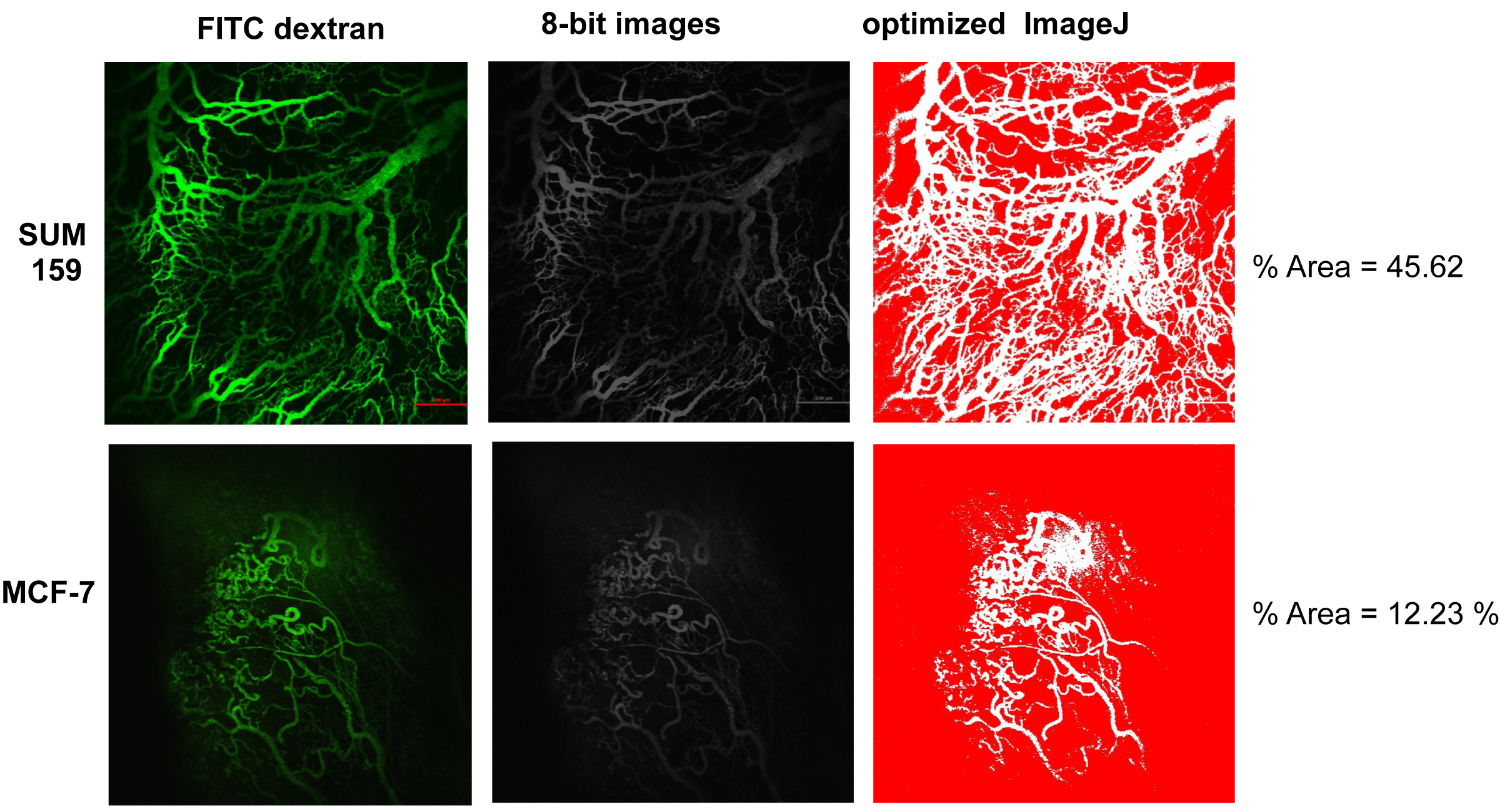

Supplement: Figure S3 — Tumor vascular analyses reveal difference in vascular characteristics in SUM159 and MCF-7 tumor cell lines. Images acquired by IVM were converted to 8-bits and vascular indices analyzed by ImageJ based on coverage of vascular tracer (70 kDa FITC-dextran). (TIF) [file pone.0086489.s003.tif]
