# Supplementary material for: Transient Mild Hyperthermia Induces E-selectin Mediated Localization of Mesoporous Silicon Vectors in Solid Tumors
Source: PLoS One. 2014 Feb 18;9(2):e86489. doi: 10.1371/journal.pone.0086489 (PMC3928046; doi:10.1371/journal.pone.0086489)
Supplement: Method S1 — Synthesis, functionalization, and characterization, and in vivo bio-distribution of gold nanorods used to generate localized MHT treatment. (DOCX) [file pone.0086489.s004.docx]

**Supplementary Information**

Transient Mild Hyperthermia Induces E-Selectin Mediated Localization of Mesoporous Silicon Vectors in Solid Tumors

Dickson K. Kirui,^a^ Juahua Mai,^a^ Anna-Lisa Palange,^a^ Guoting Qin,^a^ Anne L. van de Ven,^a^ Xuewu Liu, ^a^ Haifa Shen,^a, b^ Mauro Ferrari ^a, b, c *^

**Methods**

Synthesis and functionalization of CTAB-coated gold nanorods

CTAB-coated gold nanorods (GNRs) were prepared following a previously reported protocol [[1](#_ENREF_1)] with slight modifications. In brief, 300 mL of growth solution was prepared by mixing together 150 mL of 0.12 M CTAB (FW 364.46 g mol^-1^) and 0.15 M BDAC (Mw 396.17), 8.4 mL of 4 mM AgNO_3_ (FW 169.87 g mol^-1^), 5 mL of 15 mM HAuCl_4_ (FW 393.83 g mol^-1^) and 100 mL of Milli-Q water in a 500 mL flask. After gentle mixing and equilibration in a 27-30^o^ C oil bath, approximately 3 mL of ascorbic acid (80 mM, FW 176.12 gmoL) was added drop-wise until the mixture became colorless. After this color change, ¼th of total volume was added drop-wise was added to give a final volume of 3.75 mL. The final step was the addition of 360 µL of the seed solution to the growth solution gently stirring at 27-30^o^C. The colorless solution gradually became purple within 10-20 minutes and was allowed to grow for 40 min. After which, the solution was centrifuged at 14,000 rpm for 15 min to remove excess CTAB and BDAC surfactants. It was re-suspended and centrifuged twice to remove excess CTAB and then re-dispersed in a final volume of 120 mL Milli-Q water. The resulting CTAB-coated nanorods were characterized by UV-Vis spectrophotometric analyses which shown optimal absorbance at 805 nm and were functionalized with methyl-PEG-thiol to make them suitable for *in vivo* applications.

Preparation of Au seeds

Au particle seed solution was prepared by mixing 1 mL of 0.2M CTAB solution with HAuCl_4_ (1mL, 0.5 mM). Under gentle stirring, 120 µL of freshly prepared, ice-cold NaBH_4_ (0.01 M) was added, resulting in a brownish-yellow solution. The obtained solution was stirred for another 2 min at 25^o^ C and was used as seed solution for nanorod syntheses.

GNRs functionalization with methyl-PEG-thiol

A solution of CTAB-coated GNRs (1mL, 100 µg/mL) were gently mixed at room temperature with methyl-PEG-thiol solution (100 µL, 12. 5 mg mL^-1^) overnight and then dialyzed against ultrapure water (18 M Ω cm^-1^) using cellulose ester membrane dialysis (Spectrapor, Rancho Dominguez, CA) to drive unconjugated PEG. Dialyzed samples were filtered through 100-kDa filters (Millipore, Billerica, MA) to remove excess polymer and stored at 4^o^C. After PEG functionalization, the absorbance increased to 810 nm, which matched well with the excitation wavelength of near-infrared laser using create localized heating (Delta 30, Angio-dynamics, UK).

*In vivo* circulation and bio-distribution of PEG-coated GNRs

*In vivo* circulation duration of PEG-coated GNRs was evaluated as described previously [[2](#_ENREF_2)]. In brief, tumor-bearing mice were injected via tail vein with Au NRs in 0.15 mol/L NaCl, 0.1 mol/L Na phosphate buffer (pH 7.2; 10 mg Au/kg). At pre-determined time-points, 100 µL of blood samples were drawn periodically over 36 h from the suborbital space, diluted with PBS containing 10 mmol/L EDTA, centrifuged (4,000 rpm, 10 min) to remove RBCs, and 100 µL of supernatant collect and nanorods concentration determined by spectrophotometric analyses (DU 730, Beckman Coulter, Palo Alto, CA). The half-life was calculated to be 18 h.

For bio-distribution experiments, after vascular clearance of PEG-coated GNRs (72 h), injected animals were euthanized and organs were collected, weighed, and lyophilized for ICP-MS quantification of PEG-GNR bio-distribution.

Bio-distribution and mild hyperthermia using of passively-targeted GNRs *in vivo.*

Bio-distribution was assessed by collecting organ samples after 72-h circulation period and analyzed by inductively-coupled plasma mass spectrometry (Varian Inc., Palo Alto, CA) Samples were dissolved in aqua regia, prepared by adding 100 µl of nitric acid + 300 µl of 37% hydrochloric acid for 72 h to dissolve gold particles. Then, samples were brought up into 10 mL of 9.6 mL 2% HNO_3_ and analyzed via ICP-MS against standards. Control saline and organ samples with exogenously added PEG-NRs were utilized to calibrate the linearity of this method. All photothermal heating of GNRs was conducted at 72 h post administration (a time point after which they had completely cleared circulation) under the guidance of infrared thermography to continually illuminate the surface temperature of irradiated regions (FLIR Thermacam S60). Near infrared laser (Angio-dynamics Inc., UK) was utilized to selectively produce focal heating on tumor-bearing mice (~ 1 W/cm^2^, 810 nm) and maintained to desired peak tumor temperatures in tumor-bearing mice (~42°C for 20 min)

**References**

1. Kirui DK, Krishnan S, Strickland AD, Batt CA (2011) PAA-Derived Gold Nanorods for Cellular Targeting and Photothermal Therapy. Macromol Biosci 11: 779-788.

2. von Maltzahn G, Park J-H, Agrawal A, Bandaru NK, Das SK, et al. (2009) Computationally Guided Photothermal Tumor Therapy Using Long-Circulating Gold Nanorod Antennas. Cancer Res 69: 3892-3900.
