# Supplementary material for: Transient Mild Hyperthermia Induces E-selectin Mediated Localization of Mesoporous Silicon Vectors in Solid Tumors
Source: PLoS One. 2014 Feb 18;9(2):e86489. doi: 10.1371/journal.pone.0086489 (PMC3928046; doi:10.1371/journal.pone.0086489)
Supplement: Method S2 — Characterization of tumor cell viability by TUNEL assay and quantification of tumor vascularity with ImageJ®. (DOCX) [file pone.0086489.s005.docx]

Transient Mild Hyperthermia Induces E-Selectin Mediated Localization of Mesoporous Silicon Vectors in Solid Tumors

Dickson K. Kirui,^a^ Juahua Mai,^a^ Anna-Lisa Palange,^a^ Guoting Qin,^a^ Anne L. van de Ven,^a^ Xuewu Liu, ^a^ Haifa Shen,^a, b^ Mauro Ferrari ^a, b, c *^

**Methods**

TUNEL assay- Tumor characterization

DNA fragmentation an index of apoptosis was determined by TUNEL assay (Roche Diagnostics, Indianapolis, IN) according to the manufacturer’s specifications using paraffin sections of the breast tumors.

Tumor vascularity- Tumor characterization

Difference in tumor vascularity was confirmed by using ImageJ v1.34 (NIH, Bethesda, MD) to quantify level of vascularity on histological images. Images were converted to 8-bit, lower threshold color was adjusted to include regions with no fluorescence as a background, the upper threshold color was adjusted to include vascular region containing dye green dextran, and used to estimate the degree of tumor vascularity.
